# Supplementary material for: A pan-immunotherapy signature to predict intratumoral CD8+ T cell expansions
Source: Nat Commun. 2025 Oct 20;16:9175. doi: 10.1038/s41467-025-64107-5 (PMC12537911; doi:10.1038/s41467-025-64107-5)
Supplement: Supplementary file 1 — Supplementary Information [file 41467_2025_64107_MOESM1_ESM.pdf]

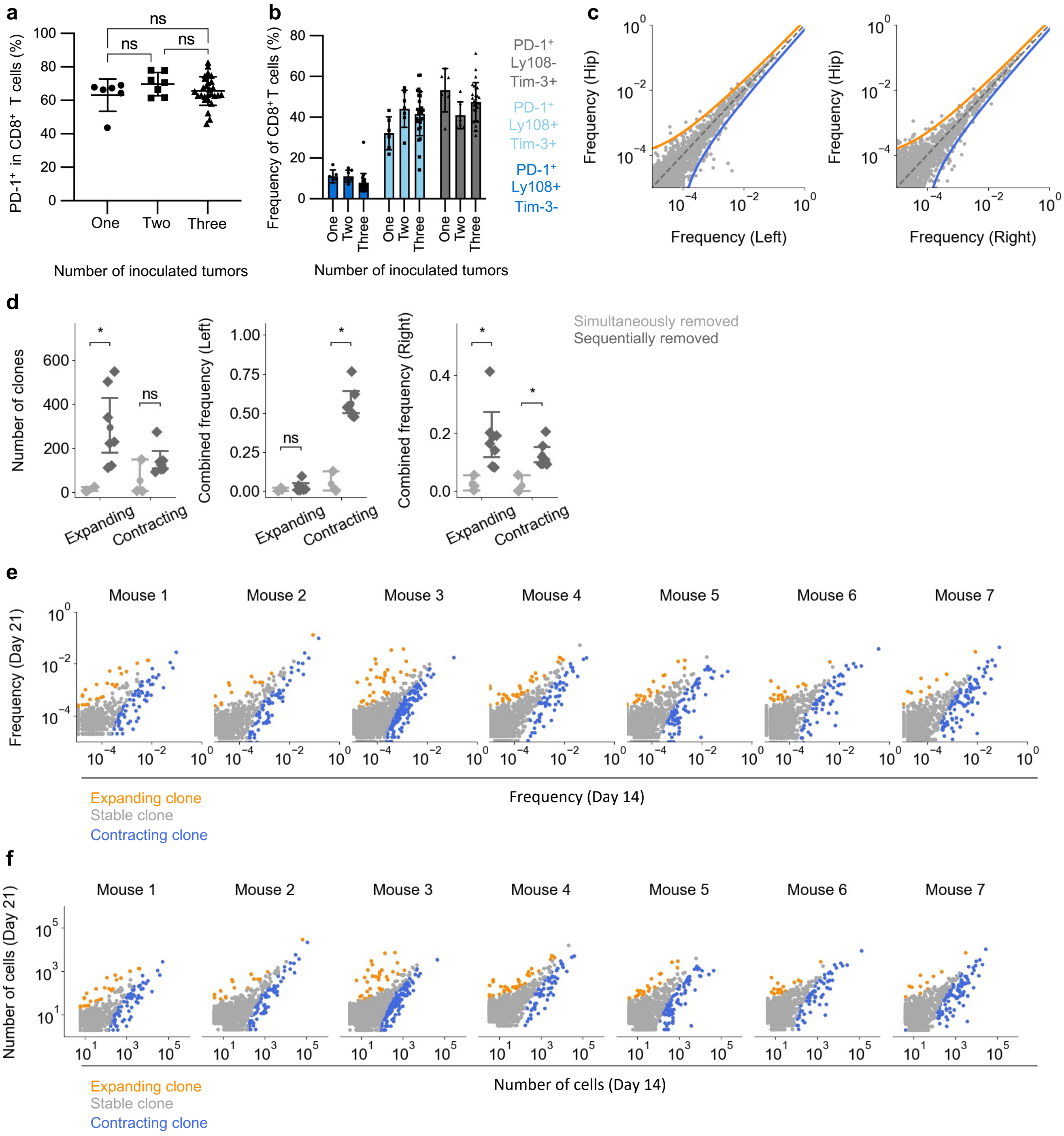

a
PD-1+ in CD8+ T cells (%)
ns
ns
ns
One
Two
Three
Number of inoculated tumors
b
Frequency of CD8+ T cells (%)
PD-1+ Ly108- Tim-3+
PD-1+ Ly108+ Tim-3+
PD-1+ Ly108+ Tim-3-
One
Two
Three
Number of inoculated tumors
c
Frequency (Hip)
Frequency (Left)
Frequency (Right)
d
Number of clones
Combined frequency (Left)
Combined frequency (Right)
Simultaneously removed
Sequentially removed
Expanding
Contracting
e
Mouse 1
Mouse 2
Mouse 3
Mouse 4
Mouse 5
Mouse 6
Mouse 7
Frequency (Day 21)
Frequency (Day 14)
Expanding clone
Stable clone
Contracting clone
f
Number of cells (Day 21)
Number of cells (Day 14)
Expanding clone
Stable clone
Contracting clone

Supplementary Fig. 1: Multi-site tumor mouse model reproducibly captures expanding and contracting clones - related to Fig. 1.

a-b) Comparing the phenotypes of $CD8^+$ T cells in tumors 14 days after mice were inoculated with different number of tumors. Fractions calculated by averaging across the tumors in each mouse. **a**) Fraction of PD-1^+ cells in $CD8^+$ T cells. **b**) Distribution of cell subsets (based on PD-1, Ly108 and TIM-3 markers) in $CD8^+$ T cells. **c**) Scatter plots displaying the frequency of clones (normalized read count of each clone) in the left flank and left hip (Left) and right flank and left hip (Right) of 3 mice excised at the same time with representative bounds defining expanding and contracting clones overlayed. **d**) The number (Left), the combined frequency in the left tumor (Middle) and the combined frequency in the right tumor (Right) of expanding and contracting clones from tumors resected simultaneously (Light grey, n=3) and sequentially on days 14 and 21 (Dark grey, n=7). **e-f**) Scatter plots displaying the frequency (**e**) and cell counts (**f**) of clones in tumors on days 14 and 21 for individual mice. Clones are colored by their expansion dynamics. Dots represent mice (**a**, **b**, **d**) and clones (**c**, **e**, **f**) with mean and standard deviation (**a**, **b**) or 95% confidence interval as shown (**d**). Statistical testing via two-sided Kruskal-Wallis test with Bonferroni correction (**a**, **d**) (****, $p<0.0001$; ***, $p<0.001$; **, $p<0.01$; *, $p<0.05$; ns $p>0.05$). Source data are provided as a Source Data file.

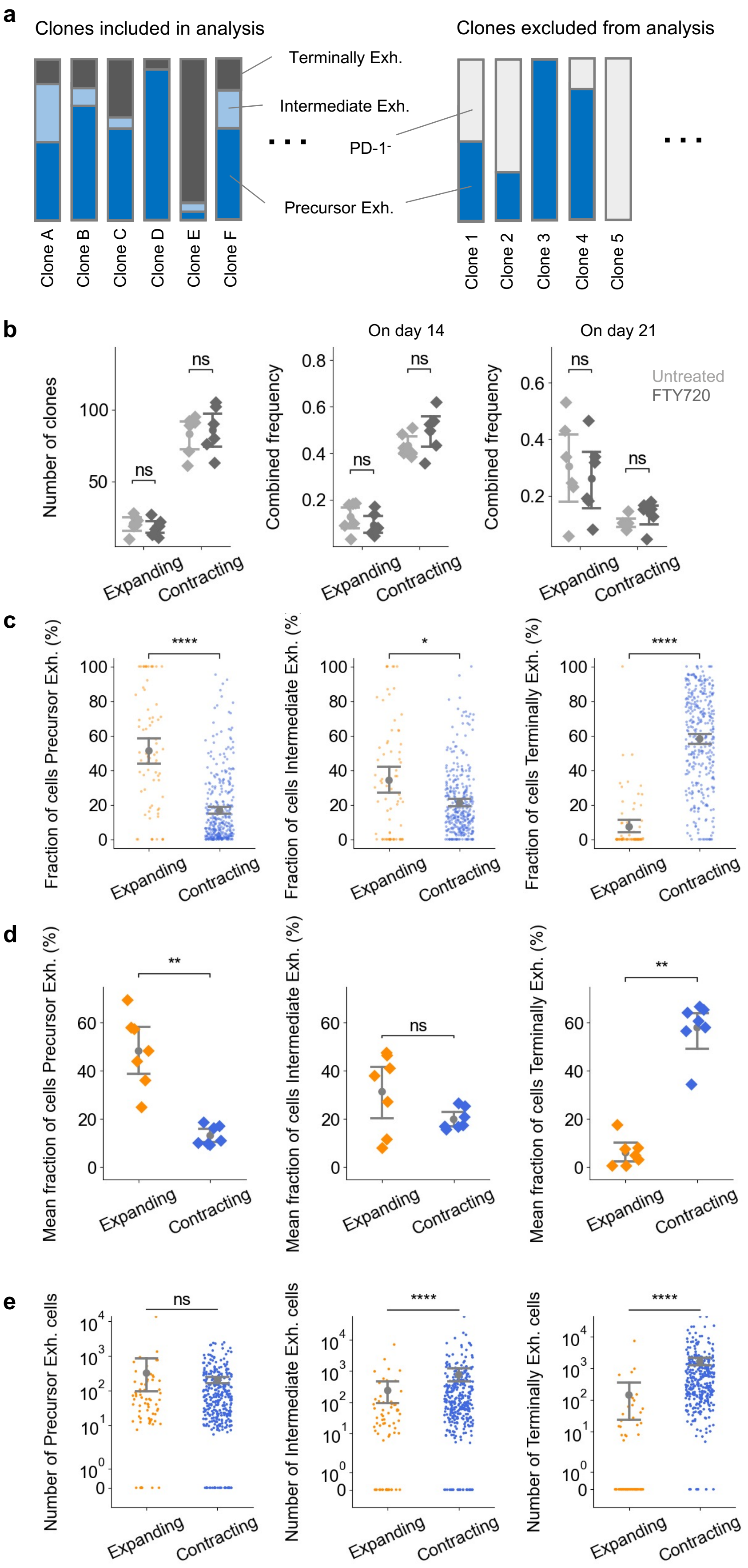
a
Clones included in analysis
Clones excluded from analysis
Terminally Exh.
Intermediate Exh.
PD-1-
Precursor Exh.
Clone A
Clone B
Clone C
Clone D
Clone E
Clone F
Clone 1
Clone 2
Clone 3
Clone 4
Clone 5
b
On day 14
On day 21
Number of clones
Combined frequency
Untreated
FTY720
ns
Expanding
Contracting
c
Fraction of cells Precursor Exh. (%)
Fraction of cells Intermediate Exh. (%)
Fraction of cells Terminally Exh. (%)
****
*
d
Mean fraction of cells Precursor Exh. (%)
Mean fraction of cells Intermediate Exh. (%)
Mean fraction of cells Terminally Exh. (%)
**
e
Number of Precursor Exh. cells
Number of Intermediate Exh. cells
Number of Terminally Exh. cells

Supplementary Fig. 2: Expanding and contracting clones have distinct cellular compositions – related to Fig. 2.

a) Diagram depicting filtering strategy used to enrich for tumor reactive clones. Merging of bulk TCR-seq datasets from sorted differentiation states enable the reconstruction of clones with their cellular compositions. Only clones with reads in the terminally exhausted dataset were selected for downstream analysis. **b**) The number (Left), the combined frequency on day 14 (Middle) and the combined frequency on day 21 (Right) of expanding and contracting clones from tumors resected simultaneously with (Dark grey, n=7) and without FTY720 between days 14 and 21 (Light grey, n=7). **c**) The fraction of precursor exhausted (Left), intermediate exhausted (Middle) and terminally exhausted (Right) cells of expanding and contracting clones. **d**) The mean fraction of precursor exhausted (Left), intermediate exhausted (Middle) and terminally exhausted (Right) cells of expanding and contracting clones in each mice. **e**) The number of precursor exhausted (Left), intermediate exhausted (Middle) and terminally exhausted (Right) cells of expanding and contracting clones. Dots represent clones (**c**, **e**) and mice (**b**, **d**) with mean and 95% confidence interval as shown (**b-e**)**.** Statistical testing via two-sided Kruskal-Wallis test (**b-e**) (****, $p<0.0001$; ***, $p<0.001$; **, $p<0.01$; *, $p<0.05$; ns $p>0.05$). Source data are provided as a Source Data file.

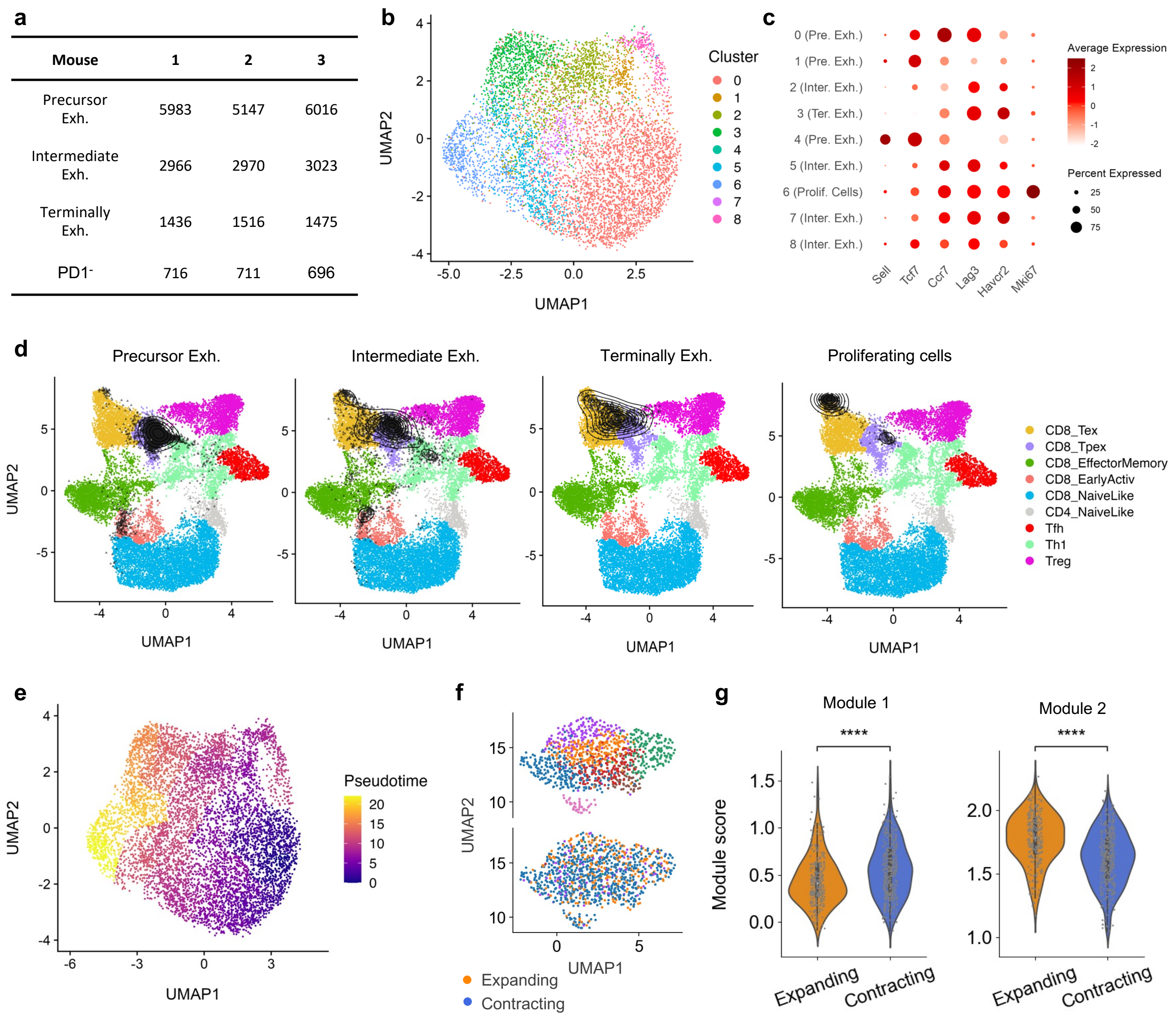

Mouse	1	2	3
Precursor Exh.	5983	5147	6016
Intermediate Exh.	2966	2970	3023
Terminally Exh.	1436	1516	1475
PD1$^-$	716	711	696

Supplementary Fig. 3: A transcriptomic atlas of intratumoral clonal dynamics – related to Fig. 3.
a) Number of cells collected for each cell type from each mouse for single cell RNA/TCR-seq analysis on day 14. The remaining cells were processed through bulk TCR-seq. **b**) UMAP map of cells colored by clusters obtained from unsupervised Louvain clustering. **c**) Dot plot showing the expression of marker genes for each cluster. **d**) Projection of cell states as defined in (**c**) to the ProjecTILs reference atlas[26]. **e**) UMAP map of cells colored by pseudo-time, calculated by Monocle 3. **f**) Re-calculated UMAP maps for precursor exhausted cells from expanding and contracting clones colored by clusters obtained from unsupervised Louvain re-clustering (Top) and clone expansion dynamics (Bottom). **g**) Precursor exhausted cells from expanding and contracting clones were scored for their expression of the Module 1 and 2 gene signatures obtained from single cell weighted gene co-expression network analysis (scWGCNA)[27,28] analysis on day 14. Dots represent genes (**c**) and cells (**b**, **d-g**). Boxplots (**g**): bottom/top of box: 25th/75th percentile; upper whisker: min(max(x), Q_3 + 1.5 * IQR), lower whisker: max(min(x), Q_1−1.5 * IQR), center: median. Statistical testing via two-sided Kruskal-Wallis test (**g**) (****, p<0.0001 ; ***, p<0.001; **, p<0.01; *, p<0.05; ns p>0.05).

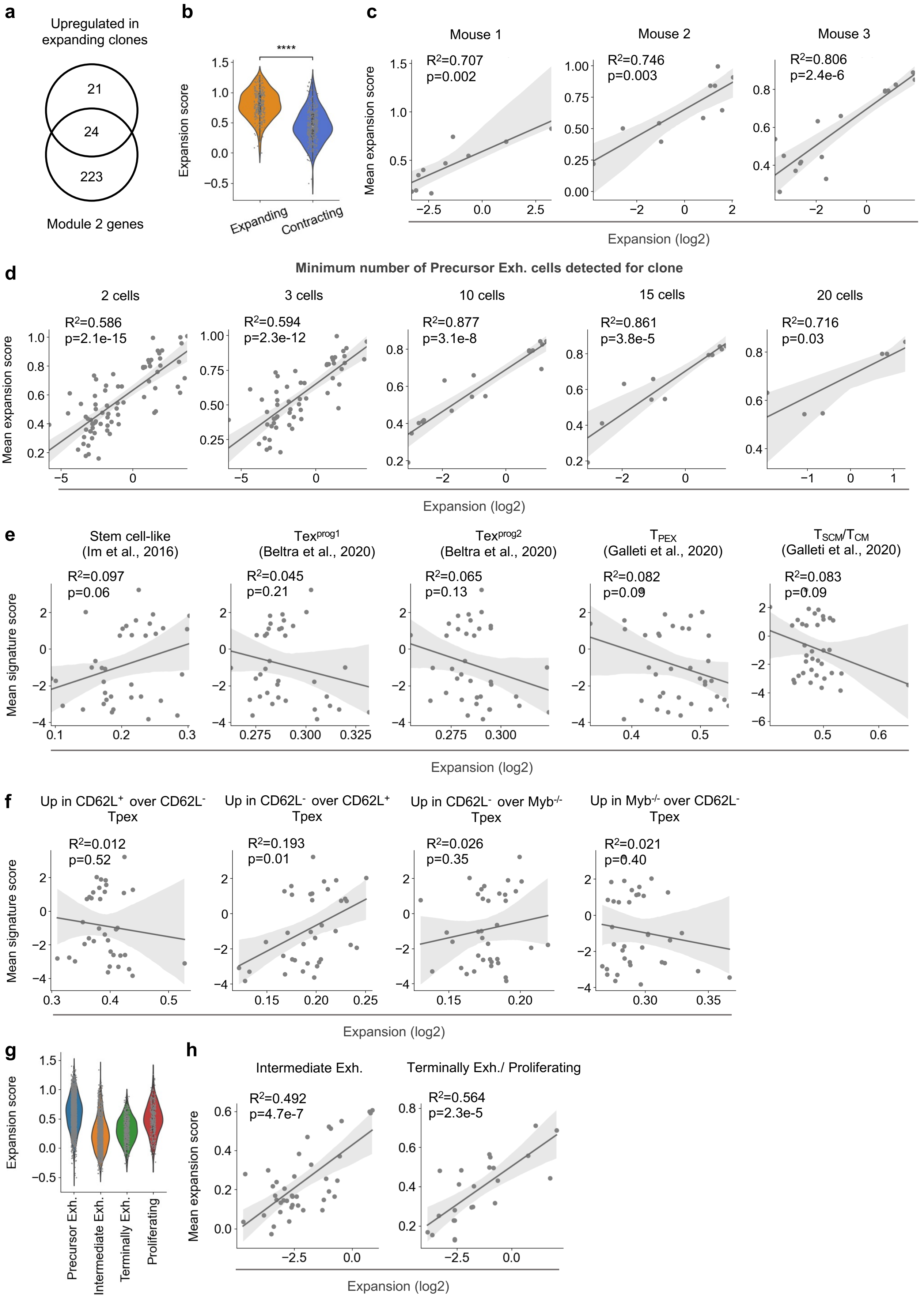
a
Upregulated in expanding clones
21
24
223
Module 2 genes
b
****
Expansion score
Expanding
Contracting
c
Mouse 1
Mouse 2
Mouse 3
R2=0.707
p=0.002
R2=0.746
p=0.003
R2=0.806
p=2.4e-6
Mean expansion score
Expansion (log2)
d
Minimum number of Precursor Exh. cells detected for clone
2 cells
3 cells
10 cells
15 cells
20 cells
R2=0.586
p=2.1e-15
R2=0.594
p=2.3e-12
R2=0.877
p=3.1e-8
R2=0.861
p=3.8e-5
R2=0.716
p=0.03
Mean expansion score
Expansion (log2)
e
Stem cell-like (Im et al., 2016)
Texprog1 (Beltra et al., 2020)
Texprog2 (Beltra et al., 2020)
TPEX (Galleti et al., 2020)
TSCM/TCM (Galleti et al., 2020)
R2=0.097
p=0.06
R2=0.045
p=0.21
R2=0.065
p=0.13
R2=0.082
p=0.09
R2=0.083
p=0.09
Mean signature score
Expansion (log2)
f
Up in CD62L+ over CD62L- Tpex
Up in CD62L- over CD62L+ Tpex
Up in CD62L- over Myb-/- Tpex
Up in Myb-/- over CD62L- Tpex
R2=0.012
p=0.52
R2=0.193
p=0.01
R2=0.026
p=0.35
R2=0.021
p=0.40
Mean signature score
Expansion (log2)
g
Expansion score
Precursor Exh.
Intermediate Exh.
Terminally Exh.
Proliferating
h
Intermediate Exh.
Terminally Exh./ Proliferating
R2=0.492
p=4.7e-7
R2=0.564
p=2.3e-5
Mean expansion score
Expansion (log2)

Supplementary Fig. 4: Expansion does not consistently correlate with previously reported precursor exhausted cell state signatures – related to Fig. 3.

a) Overlap in genes from Module 1 and genes overexpressed in cells from expanding clones. Cells (**b**) or clones (**c-d**) were scored for their expression of the expansion signature in precursor exhausted cells on day 14. **b**) Comparison of expansion signature score of cells from contracting and expanding clones. **c**) Scatter plots comparing the mean expansion signature score with the expansion (the log2 fold-increase in clone frequency from day 14 to 21) of clones for individual mice. Analysis on clones with at least 5 precursor exhausted cells on day 14. **d**) Scatter plots comparing the mean expansion signature score with the expansion of clones for different clone inclusion criteria. Inclusion criteria was altered based on the number of precursor exhausted cells in the single cell dataset for clones on day 14. **e-f**) Scatter plots comparing the mean signature score of various precursor exhausted state gene signatures (**e**) and signatures from Tsui et al., 2022[32] (**f**) with the expansion of clones. **g**) Violin plot comparing the expansion signature in different cell populations. **h**) Scatter plots comparing the mean expansion signature score of intermediate exhausted (Left) and terminally exhausted/ proliferating cells (Right) with the expansion of clones. Analysis on clones with at least 5 corresponding cells on day 14. Dots represent cells (**b**, **g**) and clones (**c-f**, **h**). Scatterplots are displayed with a line indicating the linear regression model fit and 95% confidence intervals obtained by bootstrapping (**c-f**, **h**). Boxplots (**g**): bottom/top of box: 25th/75th percentile; upper whisker: min(max(x), Q_3 + 1.5 * IQR), lower whisker: max(min(x), Q_1−1.5 * IQR), center: median. Statistical testing via two-sided Kruskal-Wallis test (**b**) and two-sided Wald test (**c-f**, **h**) (****, p<0.0001 ; ***, p<0.001; **, p<0.01; *, p<0.05; ns p>0.05). Source data are provided as a Source Data file.

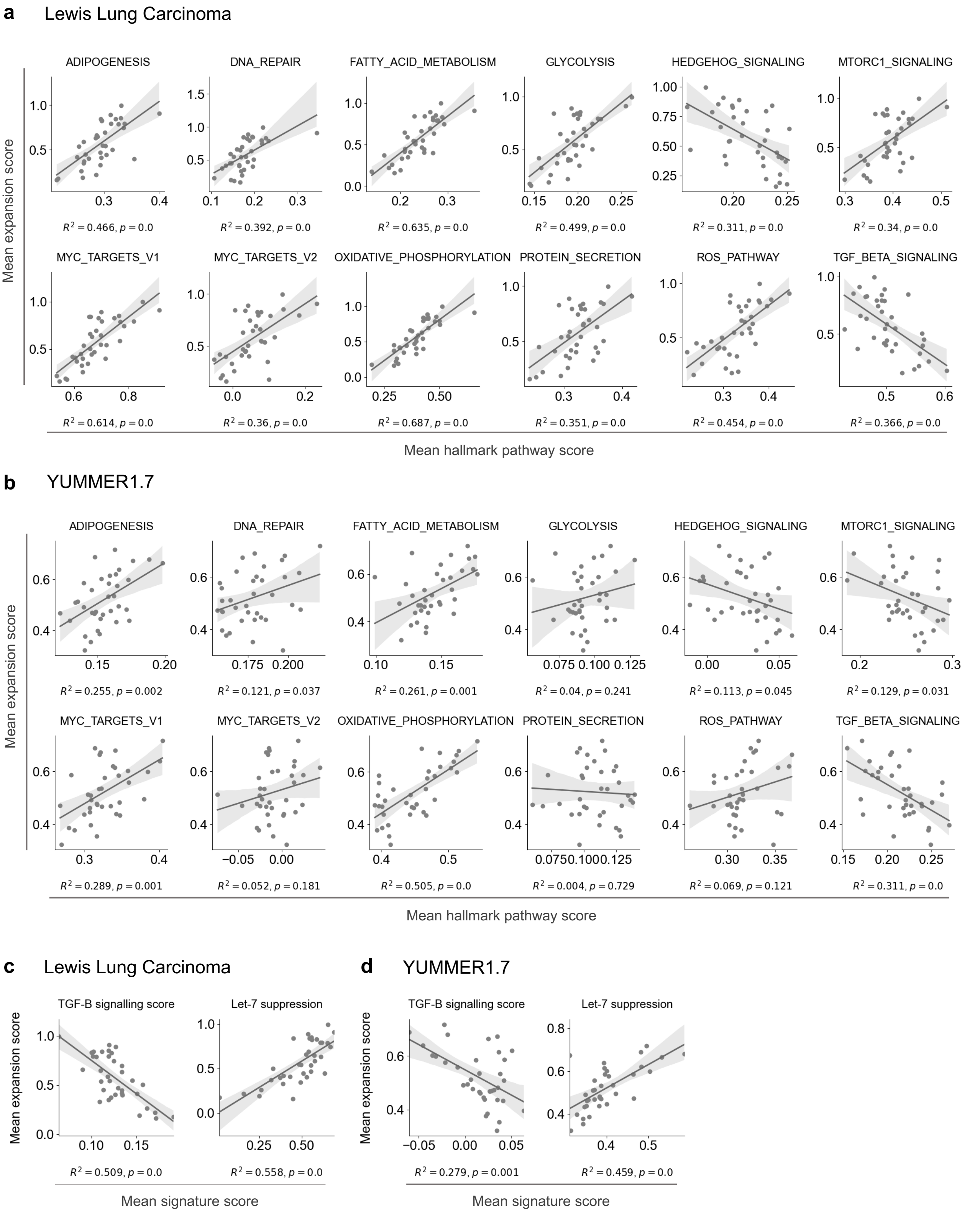
a
Lewis Lung Carcinoma
ADIPOGENESIS
DNA_REPAIR
FATTY_ACID_METABOLISM
GLYCOLYSIS
HEDGEHOG_SIGNALING
MTORC1_SIGNALING
$R^2 = 0.466, p = 0.0$
$R^2 = 0.392, p = 0.0$
$R^2 = 0.635, p = 0.0$
$R^2 = 0.499, p = 0.0$
$R^2 = 0.311, p = 0.0$
$R^2 = 0.34, p = 0.0$
MYC_TARGETS_V1
MYC_TARGETS_V2
OXIDATIVE_PHOSPHORYLATION
PROTEIN_SECRETION
ROS_PATHWAY
TGF_BETA_SIGNALING
$R^2 = 0.614, p = 0.0$
$R^2 = 0.36, p = 0.0$
$R^2 = 0.687, p = 0.0$
$R^2 = 0.351, p = 0.0$
$R^2 = 0.454, p = 0.0$
$R^2 = 0.366, p = 0.0$
Mean expansion score
Mean hallmark pathway score
b
YUMMER1.7
ADIPOGENESIS
DNA_REPAIR
FATTY_ACID_METABOLISM
GLYCOLYSIS
HEDGEHOG_SIGNALING
MTORC1_SIGNALING
$R^2 = 0.255, p = 0.002$
$R^2 = 0.121, p = 0.037$
$R^2 = 0.261, p = 0.001$
$R^2 = 0.04, p = 0.241$
$R^2 = 0.113, p = 0.045$
$R^2 = 0.129, p = 0.031$
MYC_TARGETS_V1
MYC_TARGETS_V2
OXIDATIVE_PHOSPHORYLATION
PROTEIN_SECRETION
ROS_PATHWAY
TGF_BETA_SIGNALING
$R^2 = 0.289, p = 0.001$
$R^2 = 0.052, p = 0.181$
$R^2 = 0.505, p = 0.0$
$R^2 = 0.004, p = 0.729$
$R^2 = 0.069, p = 0.121$
$R^2 = 0.311, p = 0.0$
Mean expansion score
Mean hallmark pathway score
c
Lewis Lung Carcinoma
TGF-B signalling score
Let-7 suppression
$R^2 = 0.509, p = 0.0$
$R^2 = 0.558, p = 0.0$
Mean expansion score
Mean signature score
d
YUMMER1.7
TGF-B signalling score
Let-7 suppression
$R^2 = 0.279, p = 0.001$
$R^2 = 0.459, p = 0.0$
Mean expansion score
Mean signature score

Supplementary Fig. 5: The expansion signature associates with high metabolic activity and reduced TGF-B signaling – related to Fig. 3.

a-b) Scatter plots comparing the mean expansion signature score of clones with various mean hallmark pathway signatures for our Lewis Lung Carcinoma dataset (**a**) and a YUMMER1.7 single cell dataset from Takahashi et al., 2024[38] (**b**). Analysis on clones with at least 5 precursor exhausted cells on day 14. Hallmark pathway signatures which strongly correlated ($R^2>0.3$, $p<0.05$) with the mean expansion signature score in the Lewis Lung Carcinoma dataset are shown. **c-d**) Scatter plots comparing the mean expansion signature score of clones with a T cell TGF-B signaling score (Left) and Let-7 signaling suppression score (Right) for our Lewis Lung Carcinoma dataset (**c**) and a YUMMER1.7 single cell dataset (**d**). Analysis on clones with at least 5 precursor exhausted cells on day 14. Dots represent clones (**a-d**). Scatterplots are displayed with a line indicating the linear regression model fit and 95% confidence intervals obtained by bootstrapping (**a-d**). Statistical testing via two-sided Wald test (**a-d**) (****, p<0.0001 ; ***, p<0.001; **, p<0.01; *, p<0.05; ns p>0.05). Source data are provided as a Source Data file.

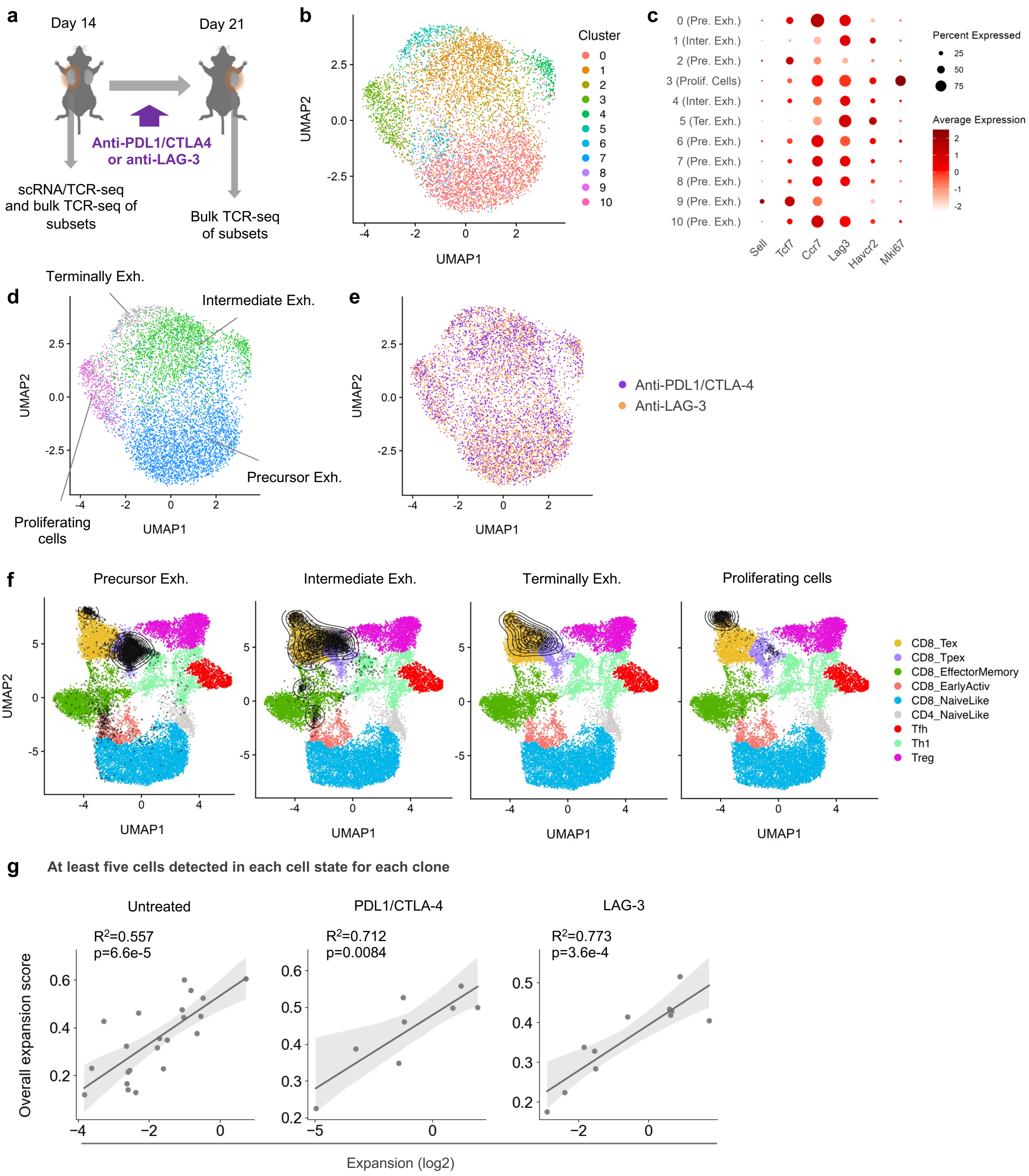

a
Day 14
Day 21
Anti-PDL1/CTLA4
or anti-LAG-3
scRNA/TCR-seq and bulk TCR-seq of subsets
Bulk TCR-seq of subsets
b
Cluster
UMAP1
UMAP2
c
0 (Pre. Exh.)
1 (Inter. Exh.)
2 (Pre. Exh.)
3 (Prolif. Cells)
4 (Inter. Exh.)
5 (Ter. Exh.)
6 (Pre. Exh.)
7 (Pre. Exh.)
8 (Pre. Exh.)
9 (Pre. Exh.)
10 (Pre. Exh.)
Sell
Tcf7
Ccr7
Lag3
Havcr2
Mki67
Percent Expressed
Average Expression
d
Terminally Exh.
Intermediate Exh.
Precursor Exh.
Proliferating cells
e
Anti-PDL1/CTLA-4
Anti-LAG-3
f
Precursor Exh.
Intermediate Exh.
Terminally Exh.
Proliferating cells
CD8_Tex
CD8_Tpex
CD8_EffectorMemory
CD8_EarlyActiv
CD8_NaiveLike
CD4_NaiveLike
Tfh
Th1
Treg
g
At least five cells detected in each cell state for each clone
Untreated
PDL1/CTLA-4
LAG-3
R²=0.557
p=6.6e-5
R²=0.712
p=0.0084
R²=0.773
p=3.6e-4
Overall expansion score
Expansion (log2)

Supplementary Fig. 6: A transcriptomic atlas of intratumoral clonal dynamics during immunotherapy – related to Fig. 4.

a) Tumors were resected from mice before and after anti-PDL1/CTLA-4 and anti-LAG-3 treatment on days 14 and 21. Day 14 samples were processed by single cell RNA/TCR-seq and the remaining cells/ samples were processed through bulk TCR-seq. **b**) UMAP map of cells colored by clusters obtained from unsupervised Louvain clustering. **c**) Dot plot showing the expression of marker genes for each cluster. **d-e**) Uniform Manifold Approximation and Projection (UMAP) map of cells from tumors, colored and labelled by cell type (**d**) and treatment (**e**). **f**) Projection of cell states as defined in (**c**) to the ProjecTILs reference atlas[26]. **g**) Scatter plots comparing the overall expansion signature score with the expansion of clones in untreated (Left), anti-PDL1/CTLA-4 (Middle) and anti-LAG-3 (Right) conditions. Analysis on clones with at least 5 cells in each cell state on day 14. Dots represent genes (**c**), cells (**b**, **d-f**) and clones (**g**). Scatterplots are displayed with a line indicating the linear regression model fit and 95% confidence intervals obtained by bootstrapping (**g**). Statistical testing via two-sided Wald test (**g**). Illustrations created with cartoons from BioRender (Ueha, S. (2025) https://BioRender.com/yx373li) (**a**). Source data are provided as a Source Data file.

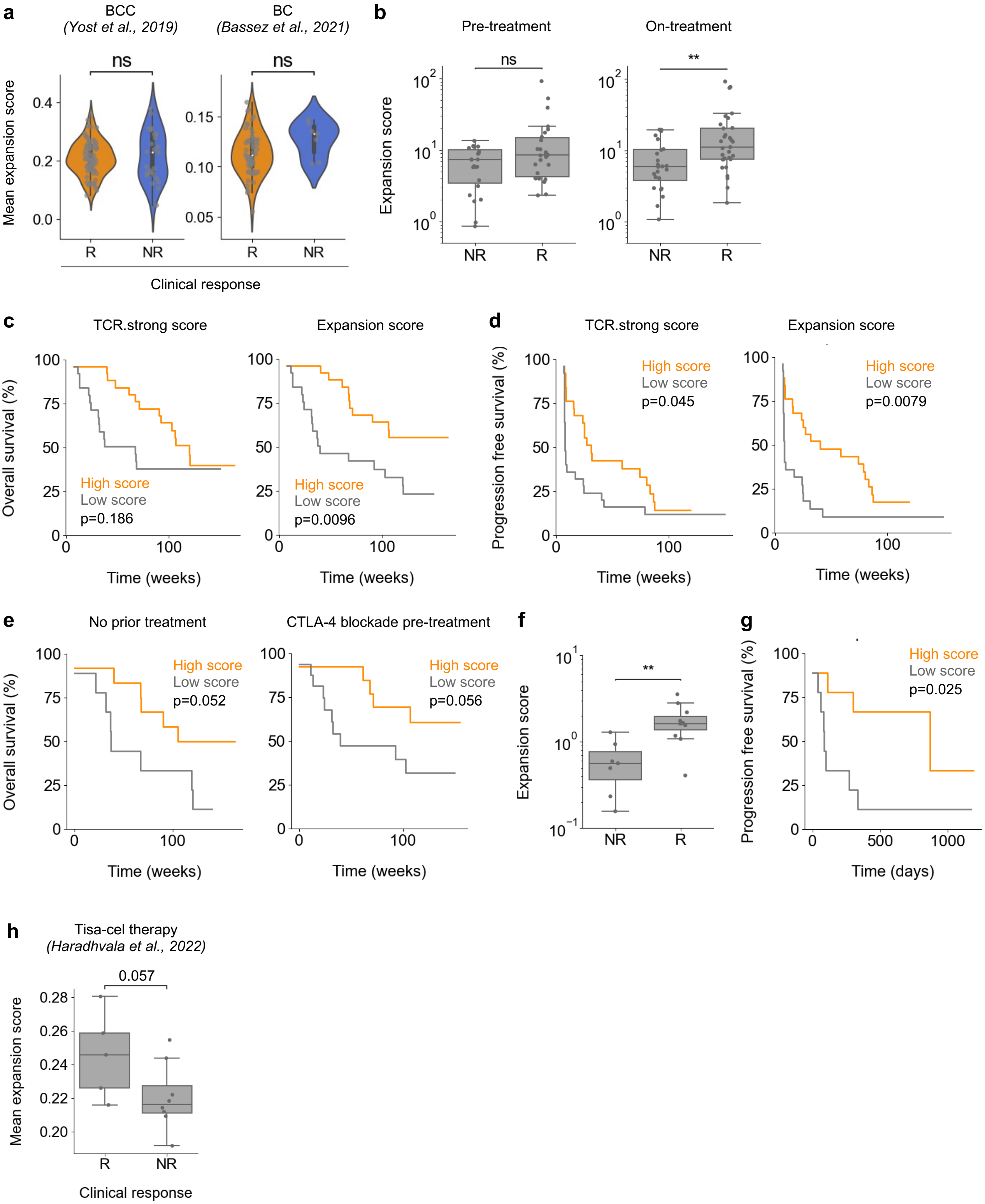
a
BCC
(Yost et al., 2019)
BC
(Bassez et al., 2021)
ns
ns
Mean expansion score
Clinical response
R
NR
b
Pre-treatment
On-treatment
ns
**
Expansion score
NR
R
c
TCR.strong score
Expansion score
Overall survival (%)
High score
Low score
p=0.186
p=0.0096
Time (weeks)
d
TCR.strong score
Expansion score
Progression free survival (%)
High score
Low score
p=0.045
p=0.0079
Time (weeks)
e
No prior treatment
CTLA-4 blockade pre-treatment
Overall survival (%)
High score
Low score
p=0.052
p=0.056
Time (weeks)
f
Expansion score
**
NR
R
g
Progression free survival (%)
High score
Low score
p=0.025
Time (days)
h
Tisa-cel therapy
(Haradhvala et al., 2022)
0.057
Mean expansion score
R
NR
Clinical response

Supplementary Fig. 7: The expansion signature stratifies melanoma patient outcomes to different immunotherapy regimens – related to Fig. 4.

a) Comparison of the mean expansion signature score of cells in clones from responding and non-responding patients before PD-1 therapy from longitudinal single-cell RNA/TCR-seq tumor biopsies from basal cell carcinoma (BCC) (Yost et al., 2019[9]) and breast cancer (BC) (Bassez et al., 2021[10]) patient datasets. **b-e**) Analysis of bulk RNA-seq tumor biopsies from patients undergoing anti-PD-1 therapy (Riaz et al., 2017[39], n=50). **b**) Comparison of expansion signature score in pre-treatment (Left) and on-treatment (Right) biopsies between non-responders (NR) and responders (R). **c-d**) Plots of Kaplan Meier estimator of overall survival (**c**) and progression free survival (**d**) stratified by TCR.stong score (above/below median) (Left, Elliot et al., 2021[40]) or by expansion signature score (above/below median) (Right) scores. **e**) Plots of Kaplan Meier estimator of overall survival stratified by expansion signature score (above/below median), splitting the cohort by their pre-treatment regimens: no prior treatment (Left, n=21) and CTLA-4 blockade pre-treatment (Right, n=29). **f-g**) Analysis of bulk RNA-seq tumor biopsies from patients undergoing anti-PD-1/CTLA-4 therapy (Gide et al., 2019[41], n=18). **f**) Comparison of expansion signature score in on-treatment biopsies between non-responders (NR) and responders (R). **g**) Plots of Kaplan Meier estimator of progression free survival (Right) stratified by expansion signature score (above/below median). **h**) Comparison of the mean expansion signature score of pre-infusion CAR-T cells between responding and non-responding patients undergoing Tisa-cel therapy (Haradhvala et al., 2022[44], n=13). Dots represent clones (**a**) and patients (**b**, **f**, **h**). Boxplots (**a**, **b**, **f**, **h**): bottom/top of box: 25th/75th percentile; upper whisker: $\min(\max(x), Q\_3 + 1.5 * IQR)$, lower whisker: $\max(\min(x), Q\_1 - 1.5 * IQR)$, center: median. Statistical testing via two-sided Kruskal-Wallis test (**a**, **b**, **f**, **h**) and two-sided Log-rank test (**c**, **d**, **e**, **g**) (****, p<0.0001 ; ***, p<0.001; **, p<0.01; *, p<0.05; ns p>0.05). Source data are provided as a Source Data file.

a

Mouse	1	2	3
Precursor Exh.	5595	5433	6989
Intermediate Exh.	1503	1508	1495
Terminally Exh.	1526	1504	1459
PD1^{-}	719	698	682

b

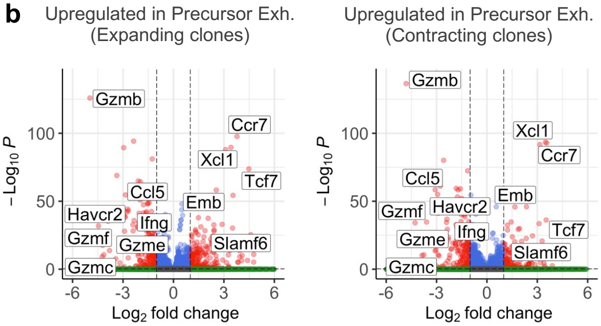

c

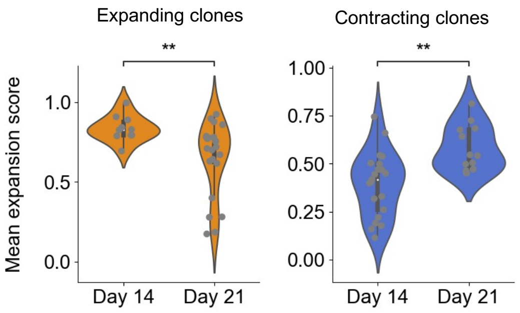

Supplementary Fig. 8: Precursor exhausted cells are maintained in the tumor during clone contraction – related to Figs. 2 and 3.

a) Number of cells collected for each cell type from each mouse for single cell RNA/TCR-seq analysis on day 21. The remaining cells were processed through bulk RNA-seq. **b**) Differential gene expression analysis of genes overexpressed in precursor exhausted cells on day 21 from expanding (Left) and contracting (Right) clones over terminally exhausted cells. Notable genes are labelled. **c**) Change in mean expansion signature score of expanding (Left) and contracting (Right) clones between day 14 and 21. Analysis on clones with at least 5 precursor exhausted cells on day 14. Dots represent genes (**b**) and clones (**c**)**.** Boxplots (**c**): bottom/top of box: 25th/75th percentile; upper whisker: min(max(x), Q_3 + 1.5 * IQR), lower whisker: max(min(x), Q_1−1.5 * IQR), center: median. Statistical testing via via two-sided Wilcoxon rank-sum test corrected with the Benjamini–Hochberg procedure (**b**) and two-sided Kruskal-Wallis test (**c**) (****, p<0.0001 ; ***, p<0.001; **, p<0.01; *, p<0.05; ns p>0.05). Source data are provided as a Source Data file.

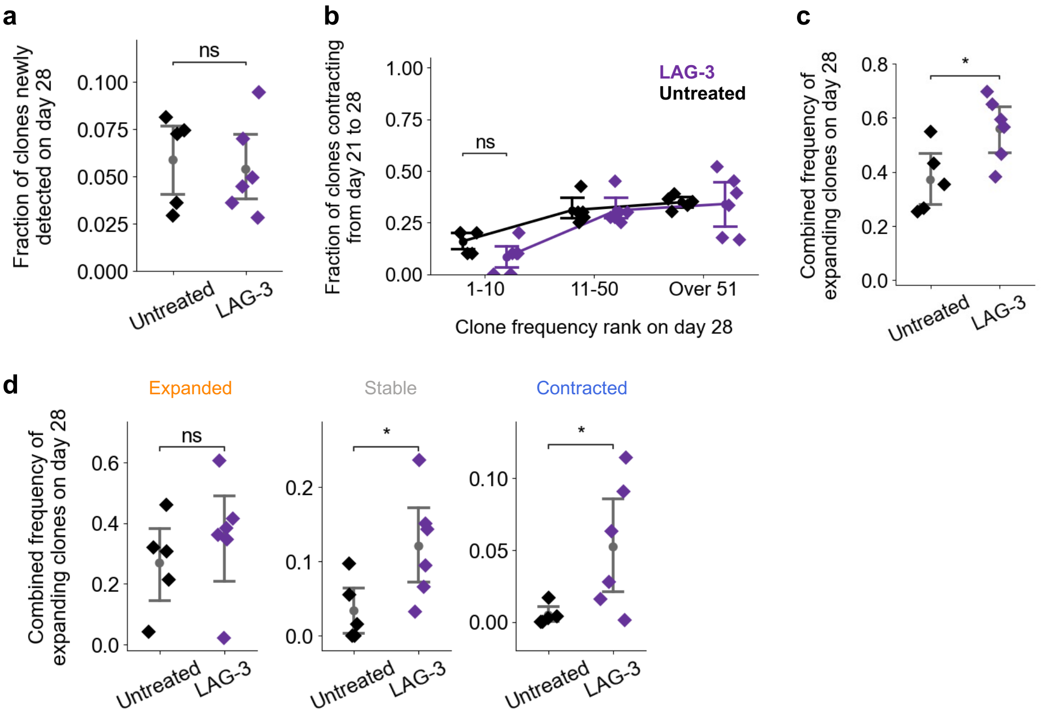

Supplementary Fig. 9: LAG-3 blockade increases the combined frequency of expanding clones - related to Fig. 5.
a) Fraction of clones detected in the tumor on day 28 that were not detected in the tumors on days 14 and 21. **b**) Fraction of clones detected in the tumor on day 28 contracting between day 21 and 28, grouped by the ranked size of the clone on day 28 and colored by each treatment regimen. **c**) Combined frequency of clones on day 28 that expanded between day 21 and 28. **d**) Combined frequency of clones expanding between day 21 to 28 on day 28 for clones that expanded (Left), were stable (Middle) or contracted (Right) between day 14 and 21 (showing top 50 clones on day 14 or 21). Dots represent mice (**a-d**) with mean and 95% confidence interval as shown (**a-d**)**.** Statistical testing via two-sided Kruskal-Wallis test (**a-d**) (****, $p<0.0001$; ***, $p<0.001$; **, $p<0.01$; *, $p<0.05$; ns $p>0.05$). Source data are provided as a Source Data file.

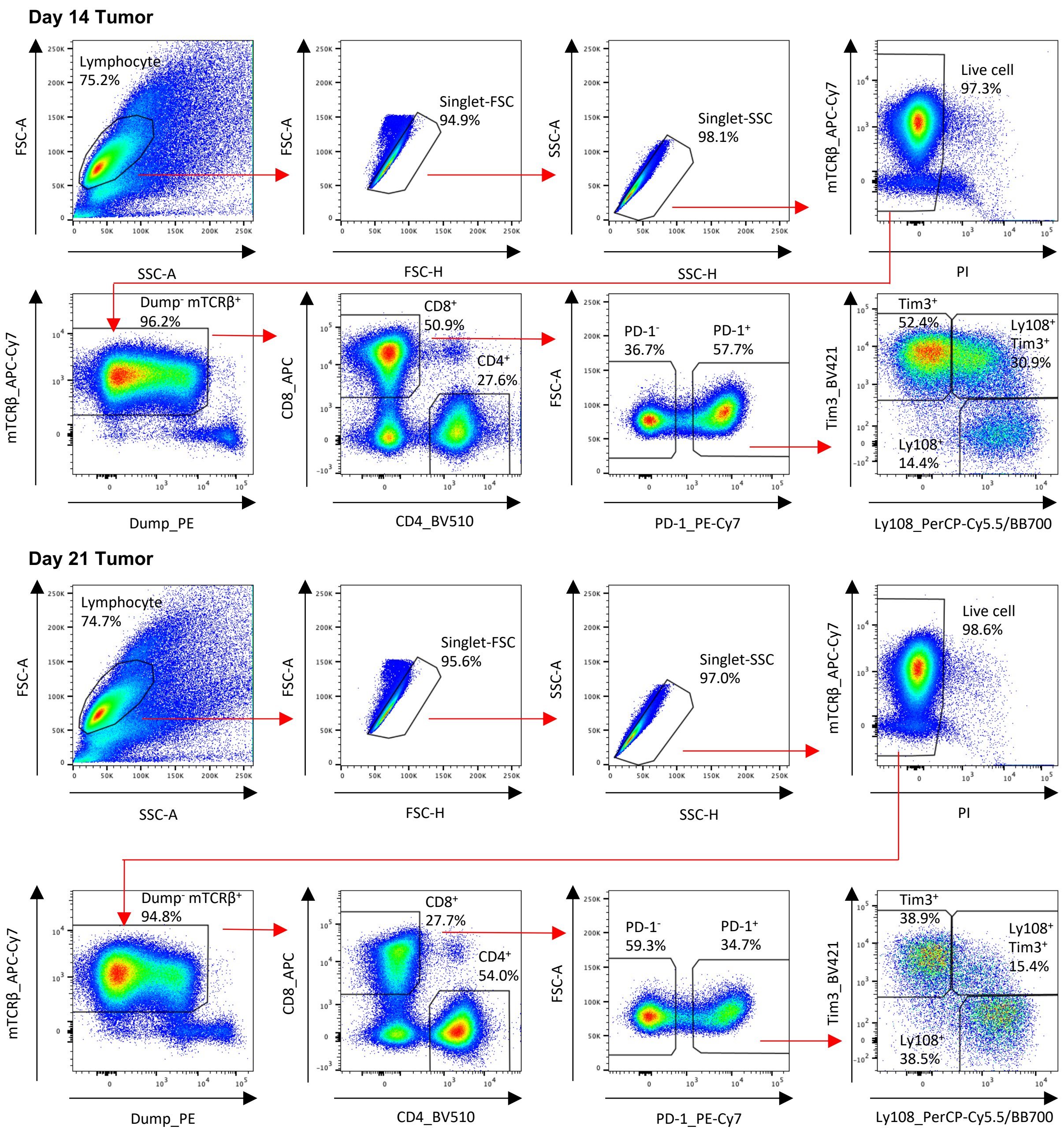

Supplementary Fig. 10: Gating strategy of tumor-infiltrating T cells from multi-site tumor-bearing mice.

Representative flow cytometry plots showing the gating strategy for TCRβ$^+$ cells enriched by magnetic separation from tumor cell suspensions prepared on days 14 and 21. Dump: CD11b, B220, NK1.1, TER119.

Supplementary Table 1: Primer sequences used for bulk TCR (top) and bulk RNA (bottom)

step	Name	Sequence	Notes	Grade
template switching	trP1-TSO	/5AmMC12/CCTCTCTATGGGCAGTCGGTGATrGrG+G		OPC
1st and 2nd WTA	5' BDWTA V2 primer	NH2-C6-AAGCAGTGGTATCAACGCAGAG		OPC
2nd WTA	illumina-i7-primer	GTGACTGGAGTTCAGACGTGTGCTCTTCCGATCT		OPC
1st WTA	illumina-i7-[BC1]-trP1	GTGACTGGAGTTCAGACGTGTGCTCTTCCGATCTAACCGCGGGGCAGTCGGTGATGGG		OPC
	illumina-i7-[BC2]-trP1	GTGACTGGAGTTCAGACGTGTGCTCTTCCGATCTGGTTATAAAGGCAGTCGGTGATGGG		OPC
	illumina-i7-[BC3]-trP1	GTGACTGGAGTTCAGACGTGTGCTCTTCCGATCTCCAAGTCCCAGGCAGTCGGTGATGGG		OPC
	illumina-i7-[BC4]-trP1	GTGACTGGAGTTCAGACGTGTGCTCTTCCGATCTTTGGACTTTCTGGCAGTCGGTGATGGG		OPC
	illumina-i7-[BC5]-trP1	GTGACTGGAGTTCAGACGTGTGCTCTTCCGATCTCAGTGGATGTATGGCAGTCGGTGATGGG		OPC
	illumina-i7-[BC6]-trP1	GTGACTGGAGTTCAGACGTGTGCTCTTCCGATCTTGACAAGCGGCAGTCGGTGATGGG		OPC
	illumina-i7-[BC7]-trP1	GTGACTGGAGTTCAGACGTGTGCTCTTCCGATCTCTAGCTTGAGGCAGTCGGTGATGGG		OPC
	illumina-i7-[BC8]-trP1	GTGACTGGAGTTCAGACGTGTGCTCTTCCGATCTTCGATCCACAGGCAGTCGGTGATGGG		OPC
	illumina-i7-[BC9]-trP1	GTGACTGGAGTTCAGACGTGTGCTCTTCCGATCTCCTGAACTTCTGGCAGTCGGTGATGGG		OPC
	illumina-i7-[BC10]-trP1	GTGACTGGAGTTCAGACGTGTGCTCTTCCGATCTTTCAGGTCGTATGGCAGTCGGTGATGGG		OPC
	illumina-i7-[BC11]-trP1	GTGACTGGAGTTCAGACGTGTGCTCTTCCGATCTAGTAGAGAGGCAGTCGGTGATGGG		OPC
	illumina-i7-[BC12]-trP1	GTGACTGGAGTTCAGACGTGTGCTCTTCCGATCTGACGAGAGAGGCAGTCGGTGATGGG		OPC
	illumina-i7-[BC13]-trP1	GTGACTGGAGTTCAGACGTGTGCTCTTCCGATCTAGACTTGGCAGGCAGTCGGTGATGGG		OPC
	illumina-i7-[BC14]-trP1	GTGACTGGAGTTCAGACGTGTGCTCTTCCGATCTGAGTCCAATCTGGCAGTCGGTGATGGG		OPC
	illumina-i7-[BC15]-trP1	GTGACTGGAGTTCAGACGTGTGCTCTTCCGATCTCTTAAGCCGTATGGCAGTCGGTGATGGG		OPC
	illumina-i7-[BC16]-trP1	GTGACTGGAGTTCAGACGTGTGCTCTTCCGATCTTCCGGATTGGCAGTCGGTGATGGG		OPC
	illumina-i7-[BC17]-trP1	GTGACTGGAGTTCAGACGTGTGCTCTTCCGATCTCTGTATTAAGGCAGTCGGTGATGGG		OPC
	illumina-i7-[BC18]-trP1	GTGACTGGAGTTCAGACGTGTGCTCTTCCGATCTTCACGCCGCAGGCAGTCGGTGATGGG		OPC
	illumina-i7-[BC19]-trP1	GTGACTGGAGTTCAGACGTGTGCTCTTCCGATCTACTTACATTCTGGCAGTCGGTGATGGG		OPC
	illumina-i7-[BC20]-trP1	GTGACTGGAGTTCAGACGTGTGCTCTTCCGATCTGTCCGTGCGTATGGCAGTCGGTGATGGG		OPC
	illumina-i7-[BC21]-trP1	GTGACTGGAGTTCAGACGTGTGCTCTTCCGATCTAAGGTACCGGCAGTCGGTGATGGG		OPC
	illumina-i7-[BC22]-trP1	GTGACTGGAGTTCAGACGTGTGCTCTTCCGATCTGGAACGTTAGGCAGTCGGTGATGGG		OPC

step	Name	Sequence	Notes	Grade
template switching	trP1-TSO	/5AmMC12/CCTCTCTATGGGCAGTCGGTGATrGrG+G		OPC
1st and 2nd WTA	5' BDWTA V2 primer	NH2-C6-AAGCAGTGGTATCAACGCAGAG		OPC
2nd WTA	illumina-i7-primer	GTGACTGGAGTTCAGACGTGTGCTCTTCCGATCT		OPC
1st WTA	illumina-i7-[BC1]-trP1	GTGACTGGAGTTCAGACGTGTGCTCTTCCGATCTAACCGCGGGGCAGTCGGTGATGGG		OPC
	illumina-i7-[BC2]-trP1	GTGACTGGAGTTCAGACGTGTGCTCTTCCGATCTGGTTATAAAGGCAGTCGGTGATGGG		OPC
	illumina-i7-[BC3]-trP1	GTGACTGGAGTTCAGACGTGTGCTCTTCCGATCTCCAAGTCCCAGGCAGTCGGTGATGGG		OPC
	illumina-i7-[BC4]-trP1	GTGACTGGAGTTCAGACGTGTGCTCTTCCGATCTTTGGACTTTCTGGCAGTCGGTGATGGG		OPC
	illumina-i7-[BC5]-trP1	GTGACTGGAGTTCAGACGTGTGCTCTTCCGATCTCAGTGGATGTATGGCAGTCGGTGATGGG		OPC
	illumina-i7-[BC6]-trP1	GTGACTGGAGTTCAGACGTGTGCTCTTCCGATCTTGACAAGCGGCAGTCGGTGATGGG		OPC
	illumina-i7-[BC7]-trP1	GTGACTGGAGTTCAGACGTGTGCTCTTCCGATCTCTAGCTTGAGGCAGTCGGTGATGGG		OPC
	illumina-i7-[BC8]-trP1	GTGACTGGAGTTCAGACGTGTGCTCTTCCGATCTTCGATCCACAGGCAGTCGGTGATGGG		OPC
	illumina-i7-[BC9]-trP1	GTGACTGGAGTTCAGACGTGTGCTCTTCCGATCTCCTGAACTTCTGGCAGTCGGTGATGGG		OPC
	illumina-i7-[BC10]-trP1	GTGACTGGAGTTCAGACGTGTGCTCTTCCGATCTTTCAGGTCGTATGGCAGTCGGTGATGGG		OPC
	illumina-i7-[BC11]-trP1	GTGACTGGAGTTCAGACGTGTGCTCTTCCGATCTAGTAGAGAGGCAGTCGGTGATGGG		OPC
	illumina-i7-[BC12]-trP1	GTGACTGGAGTTCAGACGTGTGCTCTTCCGATCTGACGAGAGAGGCAGTCGGTGATGGG		OPC
	illumina-i7-[BC13]-trP1	GTGACTGGAGTTCAGACGTGTGCTCTTCCGATCTAGACTTGGCAGGCAGTCGGTGATGGG		OPC
	illumina-i7-[BC14]-trP1	GTGACTGGAGTTCAGACGTGTGCTCTTCCGATCTGAGTCCAATCTGGCAGTCGGTGATGGG		OPC
	illumina-i7-[BC15]-trP1	GTGACTGGAGTTCAGACGTGTGCTCTTCCGATCTCTTAAGCCGTATGGCAGTCGGTGATGGG		OPC
	illumina-i7-[BC16]-trP1	GTGACTGGAGTTCAGACGTGTGCTCTTCCGATCTTCCGGATTGGCAGTCGGTGATGGG		OPC
	illumina-i7-[BC17]-trP1	GTGACTGGAGTTCAGACGTGTGCTCTTCCGATCTCTGTATTAAGGCAGTCGGTGATGGG		OPC
	illumina-i7-[BC18]-trP1	GTGACTGGAGTTCAGACGTGTGCTCTTCCGATCTTCACGCCGCAGGCAGTCGGTGATGGG		OPC
	illumina-i7-[BC19]-trP1	GTGACTGGAGTTCAGACGTGTGCTCTTCCGATCTACTTACATTCTGGCAGTCGGTGATGGG		OPC
	illumina-i7-[BC20]-trP1	GTGACTGGAGTTCAGACGTGTGCTCTTCCGATCTGTCCGTGCGTATGGCAGTCGGTGATGGG		OPC
	illumina-i7-[BC21]-trP1	GTGACTGGAGTTCAGACGTGTGCTCTTCCGATCTAAGGTACCGGCAGTCGGTGATGGG		OPC
	illumina-i7-[BC22]-trP1	GTGACTGGAGTTCAGACGTGTGCTCTTCCGATCTGGAACGTTAGGCAGTCGGTGATGGG		OPC
	illumina-i7-[BC23]-trP1	GTGACTGGAGTTCAGACGTGTGCTCTTCCGATCTAATTCTGCCAGGCAGTCGGTGATGGG		OPC
	illumina-i7-[BC24]-trP1	GTGACTGGAGTTCAGACGTGTGCTCTTCCGATCTGGCCTCATTCTGGCAGTCGGTGATGGG		OPC

Supplementary Table 2: Antibody panel and antibody information

Fluorescence	Channel (FL)	Tumor	
		Ag	Dilution
FcBlock/Monocyte block		Fc block	x100
FITC	B1		
PerCP-Cy5.5	B2	Ly108	x100
APC	R3	CD8a	x100
Alexa Fluor 700	R4		
APC-Cy7	R5	TCRb	x100
PB/BV421	V6	Tim3	x100
V500	V7	CD4	x100
BV605	V8		
BV786	V9		
PE	Y10	CD11b/B220/NK 1.1/TER119	x100
PE_594/PI	Y11	PI	x200
PE_Cy5.5/7AAD	Y12		
PE_Cy7	Y13	CD279(PD-1)	x100

Antibodies	Clone	Source	Identifier
BB700 anti-mouse Ly108	13G3	BD	Cat#742272
APC Rat Anti-Mouse CD8a	53-6.7	BD	Cat#553035
APC/Cyanine7 anti-mouse TCR β chain	H57-597	Biolegend	Cat#109220
Brilliant Violet 421™ anti-mouse CD366 (Tim-3)	RMT3-23	Biolegend	Cat#119723
Brilliant Violet 510™ anti-mouse CD4	RM4-4	Biolegend	Cat#116025
PE anti-mouse/human CD11b	M1/70	Biolegend	Cat#101208
BD Pharmingen™ PE Rat Anti-Mouse CD45R/B220	RA3-6B2	BD	Cat#561878
BD Pharmingen™ PE Mouse Anti-Mouse NK-1.1	PK136	BD	Cat#553165
BD Pharmingen™ PE Rat Anti-Mouse TER-119/Erythroid Cells	TER-119	BD	Cat#553673
PE/Cyanine7 anti-mouse CD279 (PD-1)	29F.1A12	Biolegend	Cat#135216
