# Supplementary material for: A pan-immunotherapy signature to predict intratumoral CD8+ T cell expansions
Source: Nat Commun. 2025 Oct 20;16:9175. doi: 10.1038/s41467-025-64107-5 (PMC12537911; doi:10.1038/s41467-025-64107-5)
Supplement: Supplementary file 2 — Description of Additional Supplementary Files [file 41467_2025_64107_MOESM2_ESM.pdf]

Description of Additional Supplementary Files

Supplementary Data 1 – Number of cells collected in each experiment

Supplementary Data 2 – The expansion gene signature

Supplementary Data 3 – List of gene sets used in analysis
