# Supplementary material for: A pan-immunotherapy signature to predict intratumoral CD8+ T cell expansions
Source: Nat Commun. 2025 Oct 20;16:9175. doi: 10.1038/s41467-025-64107-5 (PMC12537911; doi:10.1038/s41467-025-64107-5)
Supplement: Supplementary file 6 — Reporting Summary [file 41467_2025_64107_MOESM6_ESM.pdf]

nature portfolio

Corresponding author(s): Munetomo Takahashi, Satoshi Ueha

Last updated by author(s): Jul 17, 2025

Reporting Summary

Nature Portfolio wishes to improve the reproducibility of the work that we publish. This form provides structure for consistency and transparency in reporting. For further information on Nature Portfolio policies, see our Editorial Policies and the Editorial Policy Checklist.

Statistics

For all statistical analyses, confirm that the following items are present in the figure legend, table legend, main text, or Methods section.

n/a	Confirmed	
☐	☒	The exact sample size (*n*) for each experimental group/condition, given as a discrete number and unit of measurement
☒	☐	A statement on whether measurements were taken from distinct samples or whether the same sample was measured repeatedly
☐	☒	The statistical test(s) used AND whether they are one- or two-sided<br>*Only common tests should be described solely by name; describe more complex techniques in the Methods section.*
☒	☐	A description of all covariates tested
☐	☒	A description of any assumptions or corrections, such as tests of normality and adjustment for multiple comparisons
☐	☒	A full description of the statistical parameters including central tendency (e.g. means) or other basic estimates (e.g. regression coefficient) AND variation (e.g. standard deviation) or associated estimates of uncertainty (e.g. confidence intervals)
☐	☒	For null hypothesis testing, the test statistic (e.g. *F*, *t*, *r*) with confidence intervals, effect sizes, degrees of freedom and *P* value noted<br>*Give P values as exact values whenever suitable.*
☒	☐	For Bayesian analysis, information on the choice of priors and Markov chain Monte Carlo settings
☒	☐	For hierarchical and complex designs, identification of the appropriate level for tests and full reporting of outcomes
☐	☒	Estimates of effect sizes (e.g. Cohen's *d*, Pearson's *r*), indicating how they were calculated

Our web collection on statistics for biologists contains articles on many of the points above.

Software and code

Policy information about availability of computer code

Data collection	Flow cytometry: Data was collected on FACS Aria II or Aria III (BD Biosciences) (BD FACS Diva software version 8.0.1 or 9.0.1) or CytoFLEX S (Beckman Coulter CytExpert software version 2.3). Cell sorting was performed on FACSAria II or III using Diva software (BD ACS Diva software version 8.0.1 or 9.0.1).<br>Sequencing was performed with Novaseq 6000 S4 flow cells (Illumina).
Data analysis	Data from single cell RNA/TCR-seq and bulk RNA-seq was processed with cutadapt 2.10 and bowtie2–2.4.2.<br>Data from bulk TCR-seq was processed with Cutadapt-3.240, PRINSEQ-0.20.4 and MiXCR-3.0.5.<br>Processed data was analyzed using R version 4.0.3 and R packages (Seurat 4.0.4, edgeR 4.0.16, fGSEA 1.28.0, ProjecTILs 3.5.0, hdWGCNA 0.3.00 and monocle3 1.4.26), Python version 3.8.6 and Python packages (jupyterlab 4.1.5, numpy 1.24.4, pandas 1.5.2, scipy 1.10.1, scanpy 1.9.1, anndata 0.7.5, scikit-survival 0.22.2). Figures were produced with fGSEA 1.28.0 and EnhancedVolcano 1.20.0 in R, seaborn 0.11.0 and matplotlib 3.5.0 in Python, Prism 10, Affinity Publisher 1.10.8, FlowJo 10.10.0 and illustrations from Biorender (https://www.biorender.com/) and Irasutoya (https://www.irasutoya.com/).

For manuscripts utilizing custom algorithms or software that are central to the research but not yet described in published literature, software must be made available to editors and reviewers. We strongly encourage code deposition in a community repository (e.g. GitHub). See the Nature Portfolio guidelines for submitting code & software for further information.

Data

Policy information about availability of data

All manuscripts must include a data availability statement. This statement should provide the following information, where applicable:

- Accession codes, unique identifiers, or web links for publicly available datasets
- A description of any restrictions on data availability
- For clinical datasets or third party data, please ensure that the statement adheres to our policy

The sequencing data, and processed data generated in this study have been deposited in the Gene Expression Omnibus database database under accession code GSE303232 (control data) [https://www.ncbi.nlm.nih.gov/geo/query/acc.cgi?acc=GSE303232], GSE303087 (FTY720 data) [https://www.ncbi.nlm.nih.gov/geo/query/acc.cgi?acc=GSE303087], GSE303086 (days 14 and 21 data) [https://www.ncbi.nlm.nih.gov/geo/query/acc.cgi?acc=GSE303086], GSE303233 (days 14, 21 and 28 data) [https://www.ncbi.nlm.nih.gov/geo/query/acc.cgi?acc=GSE303233], GSE304629 (scRNA/TCR-seq untreated data) [https://www.ncbi.nlm.nih.gov/geo/query/acc.cgi?acc=GSE304629], GSE304763 (scRNA/TCR-seq immunotherapy data) [https://www.ncbi.nlm.nih.gov/geo/query/acc.cgi?acc= GSE304763] and GSE303089 (bulk RNA-seq data) [https://www.ncbi.nlm.nih.gov/geo/query/acc.cgi?acc=GSE303089]. The processed data used in this study are also available on Zenodo [https://doi.org/10.5281/zenodo.16248640]. The publicly available data used in this study are available as follows: Takahashi et al., 2024 (GSE266361 [https://www.ncbi.nlm.nih.gov/geo/query/acc.cgi?acc=GSE266361]), Yost et al., 2019 (GSE123814 [https://www.ncbi.nlm.nih.gov/geo/query/acc.cgi?acc= GSE123814]), Luoma et al., 2022 (GSE200996 [https://www.ncbi.nlm.nih.gov/geo/query/acc.cgi?acc= GSE200996]), Bassez et al., 2021 (EGAD00001006608 [https://ega-archive.org/datasets/EGAD00001006608]), Liu et al., 2021 (GSE179994 [https://www.ncbi.nlm.nih.gov/geo/query/acc.cgi?acc= GSE179994]), Haradhvala et al., 2022 (GSE197268 [https://www.ncbi.nlm.nih.gov/geo/query/acc.cgi?acc= GSE197268]), Riaz et al., 2017 (GSE91061 [https://www.ncbi.nlm.nih.gov/geo/query/acc.cgi?acc= GSE91061]) and Gide et al., 2019 (PRJEB23709 [https://www.ebi.ac.uk/ena/browser/view/PRJEB23709]). The remaining data are available within the Article, Supplementary Information or Source Data file.

Research involving human participants, their data, or biological material

Policy information about studies with human participants or human data. See also policy information about sex, gender (identity/presentation), and sexual orientation and race, ethnicity and racism.

Reporting on sex and gender	*Use the terms sex (biological attribute) and gender (shaped by social and cultural circumstances) carefully in order to avoid confusing both terms. Indicate if findings apply to only one sex or gender; describe whether sex and gender were considered in study design; whether sex and/or gender was determined based on self-reporting or assigned and methods used.*<br>*Provide in the source data disaggregated sex and gender data, where this information has been collected, and if consent has been obtained for sharing of individual-level data; provide overall numbers in this Reporting Summary. Please state if this information has not been collected.*<br>*Report sex- and gender-based analyses where performed, justify reasons for lack of sex- and gender-based analysis.*
Reporting on race, ethnicity, or other socially relevant groupings	*Please specify the socially constructed or socially relevant categorization variable(s) used in your manuscript and explain why they were used. Please note that such variables should not be used as proxies for other socially constructed/relevant variables (for example, race or ethnicity should not be used as a proxy for socioeconomic status).*<br>*Provide clear definitions of the relevant terms used, how they were provided (by the participants/respondents, the researchers, or third parties), and the method(s) used to classify people into the different categories (e.g. self-report, census or administrative data, social media data, etc.)*<br>*Please provide details about how you controlled for confounding variables in your analyses.*
Population characteristics	*Describe the covariate-relevant population characteristics of the human research participants (e.g. age, genotypic information, past and current diagnosis and treatment categories). If you filled out the behavioural & social sciences study design questions and have nothing to add here, write "See above."*
Recruitment	*Describe how participants were recruited. Outline any potential self-selection bias or other biases that may be present and how these are likely to impact results.*
Ethics oversight	*Identify the organization(s) that approved the study protocol.*

Note that full information on the approval of the study protocol must also be provided in the manuscript.

Field-specific reporting

Please select the one below that is the best fit for your research. If you are not sure, read the appropriate sections before making your selection.

[x] Life sciences [ ] Behavioural & social sciences [ ] Ecological, evolutionary & environmental sciences

For a reference copy of the document with all sections, see nature.com/documents/nr-reporting-summary-flat.pdf

Life sciences study design

All studies must disclose on these points even when the disclosure is negative.

Sample size	Sample size was determined on previous studies, including ours: PMID 34759926
Data exclusions	Mice with incomplete excision resulting in immediate (<7 days) regrowth of excised tumors, or tumors that did not grow (<100mm3 by day

	28) were excluded from the analysis.
Replication	The number of biological replicates is indicated in each figure legend.
Randomization	Mice were indiscriminately allocated into experimental groups.
Blinding	No blinding was used as no subjective scoring methods were used.

Reporting for specific materials, systems and methods

We require information from authors about some types of materials, experimental systems and methods used in many studies. Here, indicate whether each material, system or method listed is relevant to your study. If you are not sure if a list item applies to your research, read the appropriate section before selecting a response.

Materials & experimental systems

n/a	Involved in the study	
☐	☒	Antibodies
☐	☒	Eukaryotic cell lines
☒	☐	Palaeontology and archaeology
☐	☒	Animals and other organisms
☒	☐	Clinical data
☒	☐	Dual use research of concern
☒	☐	Plants

Methods

n/a	Involved in the study	
☒	☐	ChIP-seq
☐	☒	Flow cytometry
☒	☐	MRI-based neuroimaging

Antibodies

Antibodies used	For flow cytometry/ sorting:<br>BB700 anti-mouse Ly108 13G3 BD Cat#742272<br>APC Rat Anti-Mouse CD8a 53-6.7 BD Cat#553035<br>APC/Cyanine7 anti-mouse TCR β chain H57-597 Biolegend Cat#109220<br>Brilliant Violet 421™ anti-mouse CD366 (Tim-3) RMT3-23 Biolegend Cat#119723<br>Brilliant Violet 510™ anti-mouse CD4 RM4-4 Biolegend Cat#116025<br>PE anti-mouse/human CD11b M1/70 Biolegend Cat#101208<br>BD Pharmingen™ PE Rat Anti-Mouse CD45R/B220 RA3-6B2 BD Cat#561878<br>BD Pharmingen™ PE Mouse Anti-Mouse NK-1.1 PK136 BD Cat#553165<br>BD Pharmingen™ PE Rat Anti-Mouse TER-119/Erythroid Cells TER-119 BD Cat#553673<br>PE/Cyanine7 anti-mouse CD279 (PD-1) 29F.1A12 Biolegend Cat#135216<br>For treatment, anti-LAG-3 (clone C9B7W, BioLegend or Selleck), anti-PD-L1 (clone 10F.9G2, BioLegend) and anti-CTLA-4 (clone 9D9, Bioxcell).
Validation	Commercially available antibodies were validated by their respective manufacturers.

Eukaryotic cell lines

Policy information about cell lines and Sex and Gender in Research

Cell line source(s)	Lewis lung carcinoma (LLC) cell line was provided by Nihonkayaku (Tokyo, Japan)
Authentication	Cell lines used in this study were not authenticated.
Mycoplasma contamination	The cell lines has been confirmed to be free of Mycoplasma contamination.
Commonly misidentified lines<br>(See ICLAC register)	No commonly misidentified cell lines were used in this study.

Animals and other research organisms

Policy information about studies involving animals; ARRIVE guidelines recommended for reporting animal research, and Sex and Gender in Research

Laboratory animals	Eight-week-old female C57BL/6 mice (CD90.2, RRID: MGI:5488963) were purchased from Sankyo Labo Service Corporation Inc. All mice were bred at specific pathogen-free facilities at Tokyo University of Science and housed under a 12-hour light/dark cycle at a controlled room temperature of 23±2°C and relative humidity of 55±10%.
Wild animals	No wild animals were used in the study.

Reporting on sex | We chose to use female mice in these studies as in previous studies, including ours: PMID 34759926

Field-collected samples | Field-collected samples were not used in this study.

Ethics oversight | All animal experiments were conducted in accordance with institutional guidelines with the approval of the Animal Care and Use Committee of Tokyo University of Science. The maximum tumor size allowed by the institutional ethical board was 20mm diameter or 10% of body weight, and this was not exceeded.

Note that full information on the approval of the study protocol must also be provided in the manuscript.

Plants

Seed stocks | *Report on the source of all seed stocks or other plant material used. If applicable, state the seed stock centre and catalogue number. If plant specimens were collected from the field, describe the collection location, date and sampling procedures.*

Novel plant genotypes | *Describe the methods by which all novel plant genotypes were produced. This includes those generated by transgenic approaches, gene editing, chemical/radiation-based mutagenesis and hybridization. For transgenic lines, describe the transformation method, the number of independent lines analyzed and the generation upon which experiments were performed. For gene-edited lines, describe the editor used, the endogenous sequence targeted for editing, the targeting guide RNA sequence (if applicable) and how the editor was applied.*

Authentication | *Describe any authentication procedures for each seed stock used or novel genotype generated. Describe any experiments used to assess the effect of a mutation and, where applicable, how potential secondary effects (e.g. second site T-DNA insertions, mosiacism, off-target gene editing) were examined.*

Flow Cytometry

Plots

Confirm that:

☐ The axis labels state the marker and fluorochrome used (e.g. CD4-FITC).

☐ The axis scales are clearly visible. Include numbers along axes only for bottom left plot of group (a 'group' is an analysis of identical markers).

☐ All plots are contour plots with outliers or pseudocolor plots.

☐ A numerical value for number of cells or percentage (with statistics) is provided.

Methodology

Sample preparation | Cells were stained with a mix of Fc Block (anti-mouse CD16/CD32 mAb; clone 2.4G2, BioLegend) and fluorophore-conjugated anti-mouse monoclonal antibodies (Supplementary Table 5). Antibody mixtures were prepared using BD Horizon Brilliant Stain Buffer Plus (BD) diluted 5x with FACS buffer (D-PBS(-) supplemented with 2% FBS, and 0.05% Sodium Azide). Staining was performed for 20 minutes at 4°C. In the single-cell RNA-seq/TCR-seq experiment, cells were stained with antibodies and anti-CD45 Sample Tag oligonucleotide-conjugated antibodies from the Single-Cell Multiplexing Kit (BD Biosciences). Before sorting, T cells were enriched from tumor suspensions by further staining with BD IMag APC Magnetic Particles-DM (BD Biosciences), then placed on magnetic plate. After washing by MACS buffer (D-PBS(-) supplemented with 10% BSA, and 2 mM EDTA pH8.0), APC and APC-Cy7(CD8, mouse TCRβ)-positive cells were collected as positive fraction.

Instrument | Data was collected on FACS Aria II or Aria III (BD Biosciences) (BD FACS Diva software version 8.0.1 or 9.0.1) or CytoFLEX S (Beckman Coulter CytExpert software version 2.3). Cell sorting was performed on FACSAria II or III using Diva software (BD ACS Diva software version 8.0.1 or 9.0.1).

Software | Data was analyzed with FlowJo 10.10.0.

Cell population abundance | Although we did not confirm the purity of the samples sorted directly into lysis buffer in this analysis, for cases where distinct populations such as CD4/CD8 are clearly separated and re-analyzed after sorting, we have confirmed that the purity of the population is 98% or higher, and contamination from other subsets is less than 0.1%.

Gating strategy | Nonviable cells were excluded from the analysis based on forward and side scatter profiles and PI staining.For the bulk TCR-seq experiments, cells were sorted from the CD8+, CD8+PD-1+Ly108+TIM-3-, CD8+PD-1+Ly108+TIM-3+, CD8+PD-1+Ly108-TIM-3+ and CD8+PD-1- fractions to produce five datasets per mouse. For the single-cell RNA/TCR-seq and bulk TCR-seq experiment, cells were sorted from the CD8+PD-1+Ly108+TIM-3-, CD8+PD-1+Ly108+TIM-3+, CD8+PD-1+Ly108-TIM-3+ and CD8+PD-1- fractions. From each of the fractions, a fixed number of cells was aliquoted for single cell sequencing, and the rest was processed by bulk TCR-seq. The numbers of cells collected for each data in each analysis are shown in Supplementary Table 1. For bulk RNA-seq samples, cells were sorted from the CD8+PD-1+Ly108+TIM-3-CD62L- fraction.

☒ Tick this box to confirm that a figure exemplifying the gating strategy is provided in the Supplementary Information.
